# Supplementary material for: Influence of Graft Positioning during the Latarjet Procedure on Shoulder Stability and Articular Contact Pressure: Computational Analysis of the Bone Block Effect
Source: Biology (Basel). 2022 Dec 8;11(12):1783. doi: 10.3390/biology11121783 (PMC9775173; doi:10.3390/biology11121783)
Supplement: Supplementary file 1 [file biology-11-01783-s001.zip › biology-2038989-SI.pdf]

1. Contact pressure distribution for the 0º shoulder abduction in the scapular plane in neutral rotation

For the sake of comparison, Figures S1 and S3 present the contact pressures for the different bone graft positions under a similar scale. For a more detailed evaluation of the results obtained for each bone graft position, Figures S2 and S4 present the same contact pressures considering scales adjusted for each model.

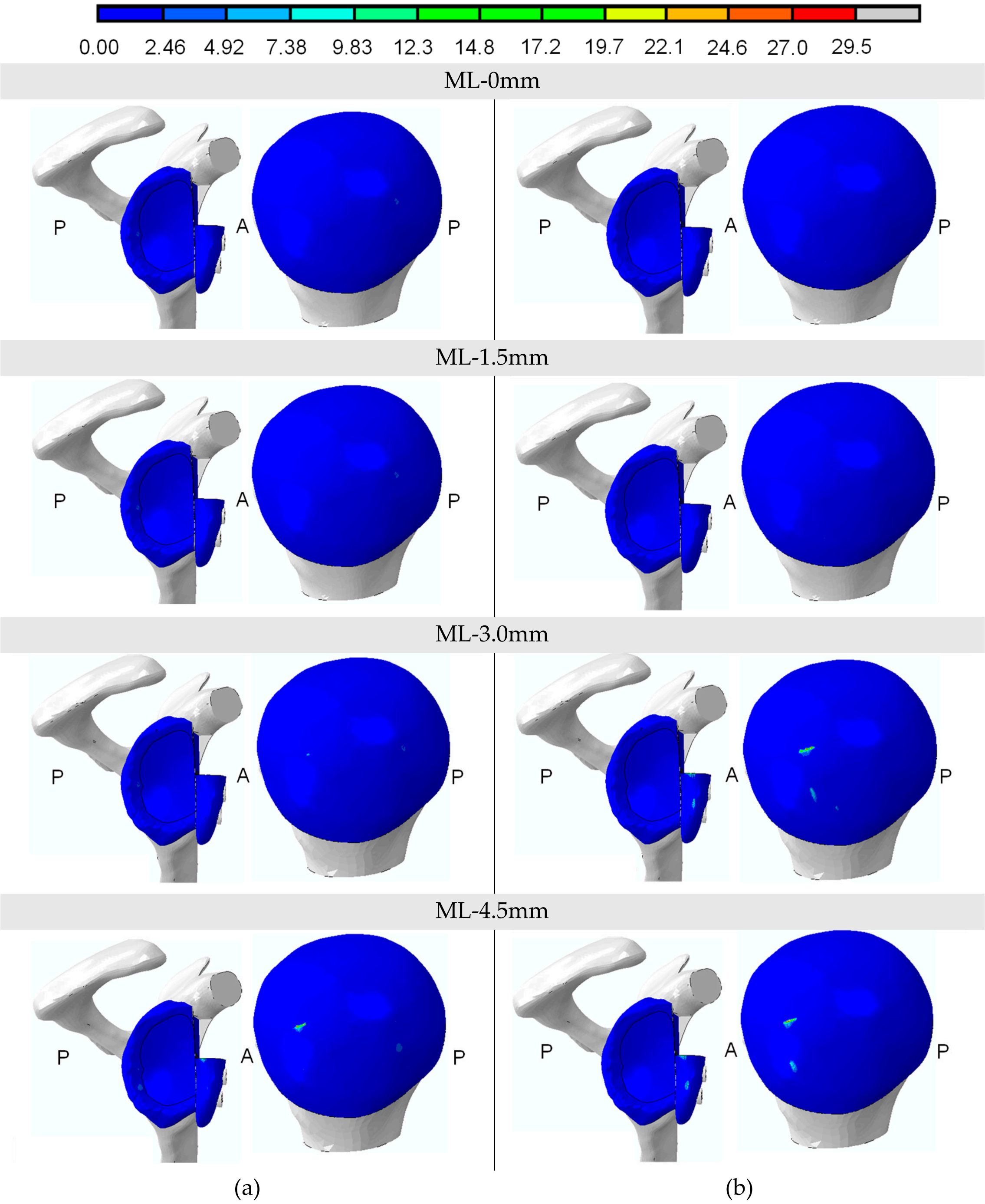

Figure S1 – Contact pressure distribution, in MPa, after the Latarjet procedure for two anterior translation points: (a) at the beginning, and (b) at the peak translation force. Results correspond to the 0º shoulder abduction in the scapular plane in neutral rotation under a 50 N compressive force. A similar scale is considered for all bone graft positions.

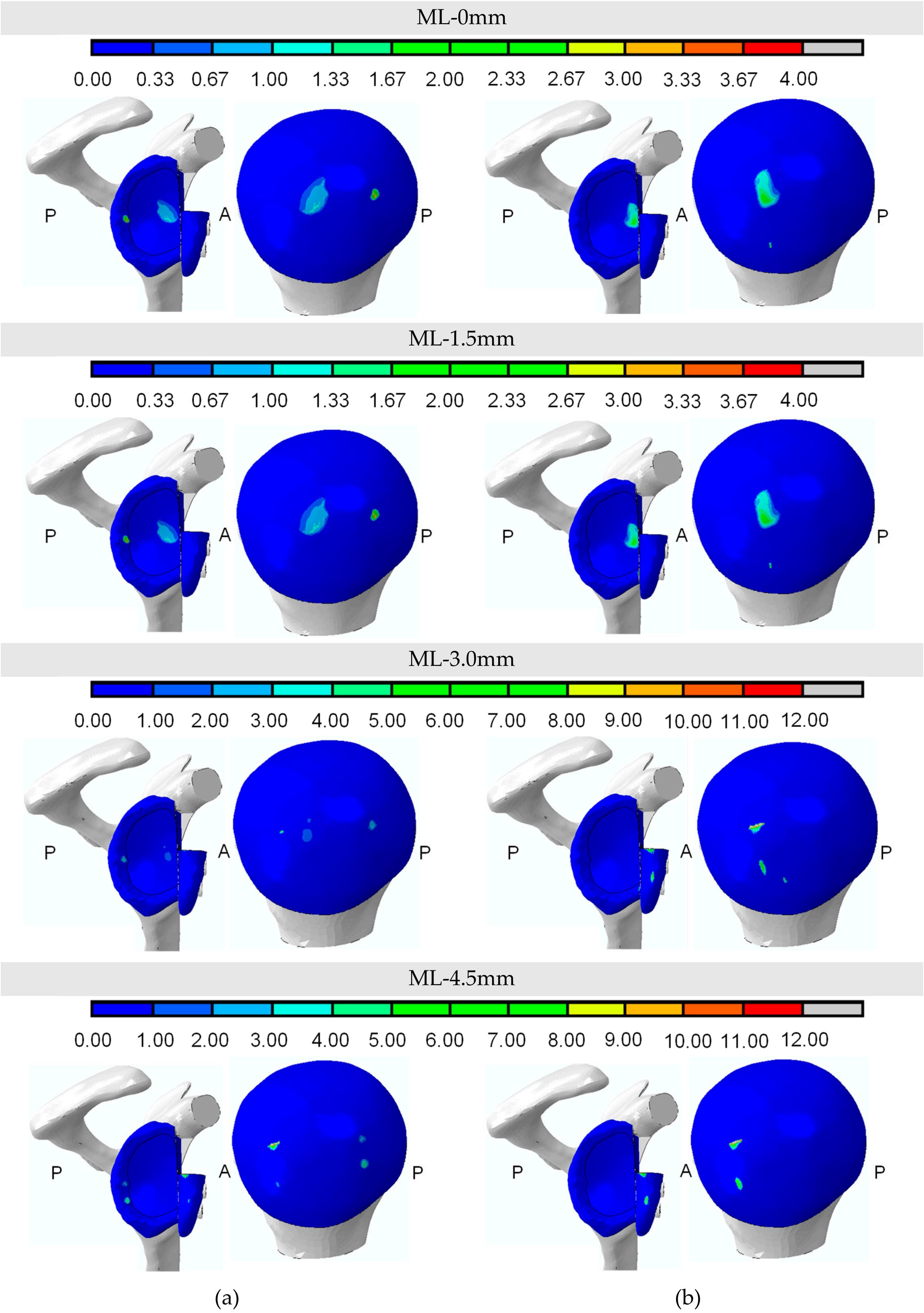

Figure S2 – Contact pressure distribution, in MPa, after the Latarjet procedure for two anterior translation points: (a) at the beginning, and (b) at the peak translation force. Results correspond to the 0º shoulder abduction in the scapular plane in neutral rotation under a 50 N compressive force. Each bone graft position presents its own scale.

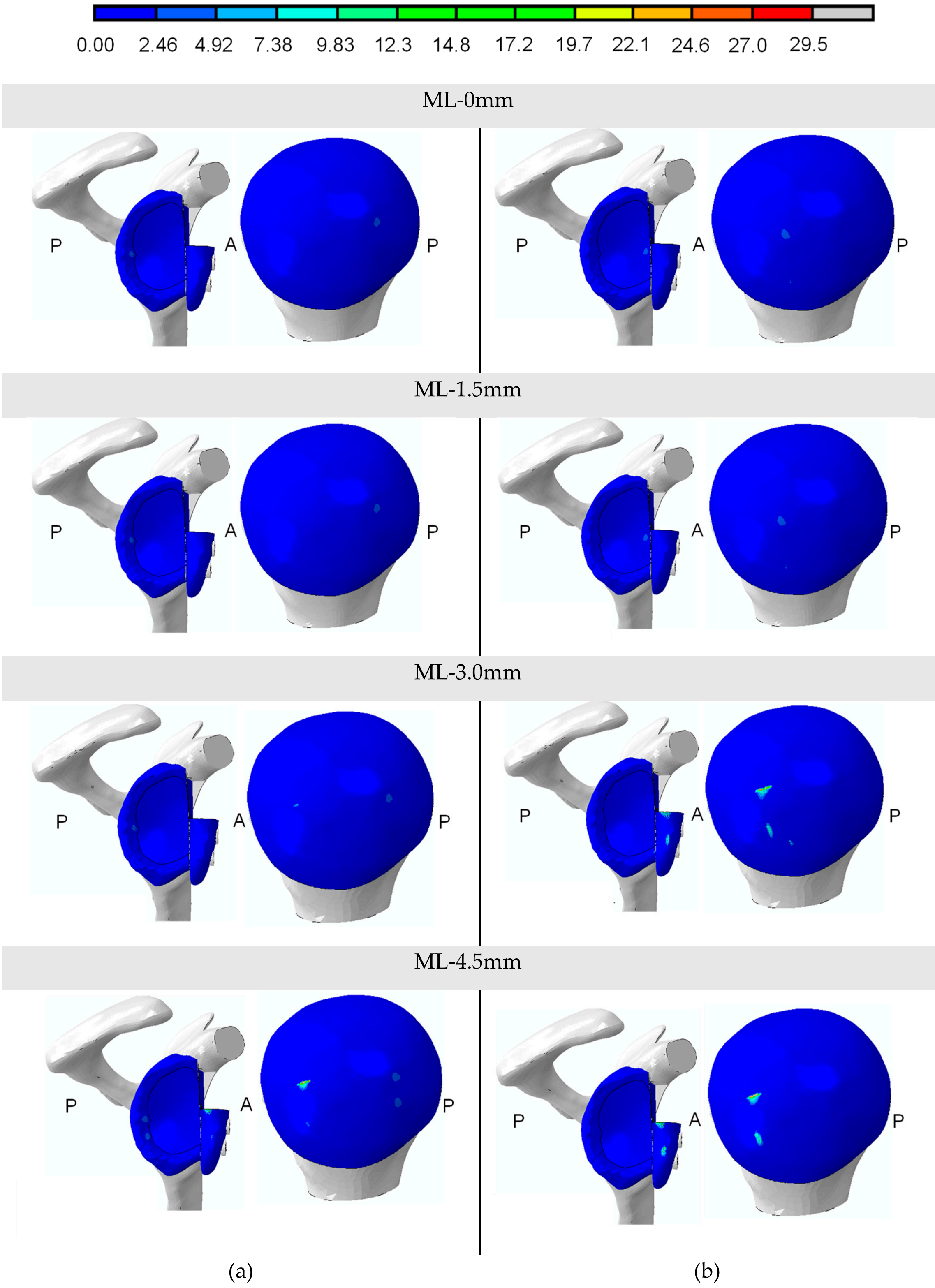

Figure S3 – Contact pressure distribution, in MPa, after the Latarjet procedure for two anterior translation points: (a) at the beginning, and (b) at the peak translation force. Results correspond to the 0º shoulder abduction in the scapular plane in neutral rotation under a 100 N compressive force. A similar scale is considered for all bone graft positions.

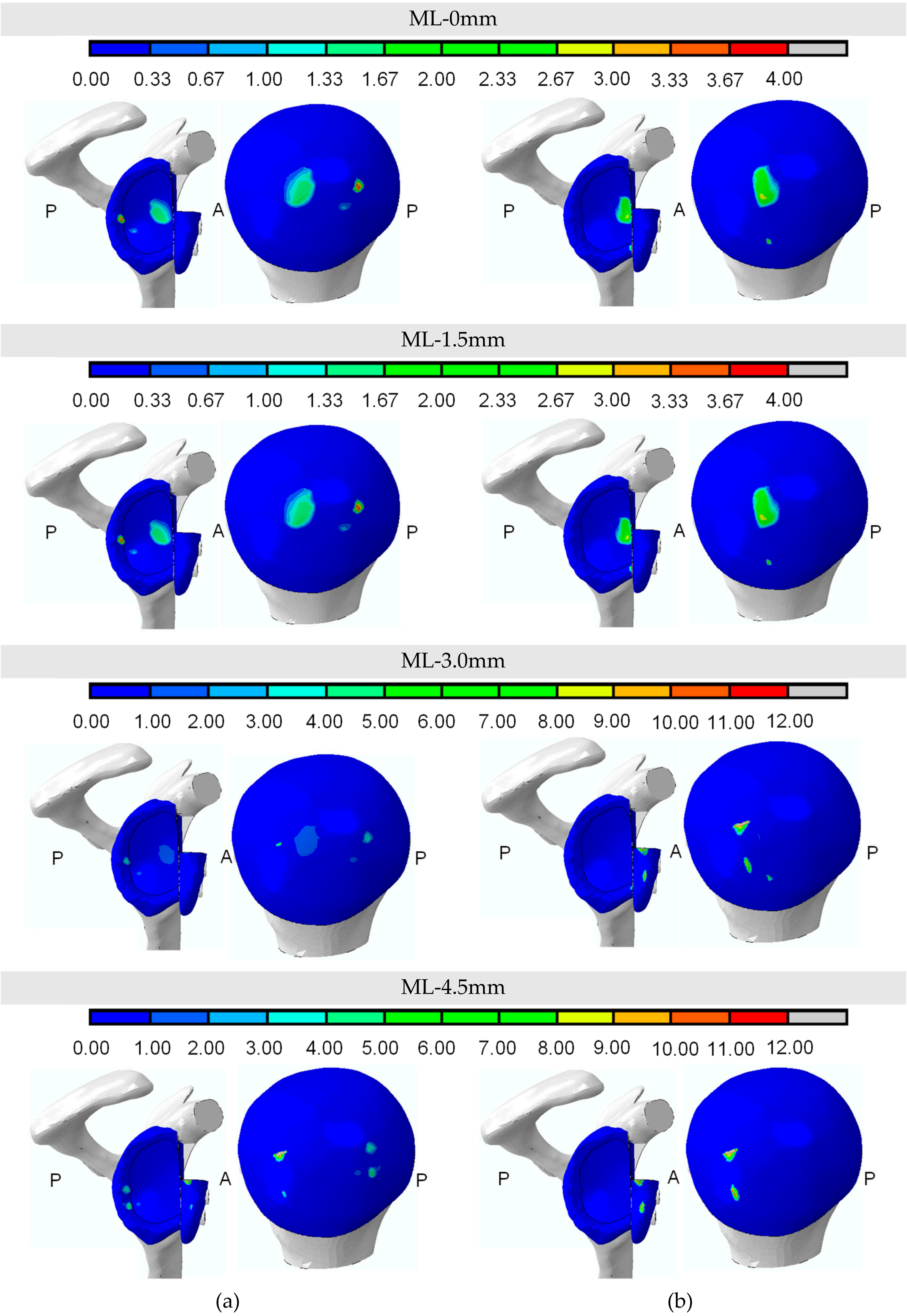

Figure S4 – Contact pressure distribution, in MPa, after the Latarjet procedure for two anterior translation points: (a) at the beginning, and (b) at the peak translation force. Results correspond to the 0º shoulder abduction in the scapular plane in neutral rotation under a 100 N compressive force. Each bone graft position presents its own scale.

2. Contact pressure distribution for the 60º shoulder abduction in the scapular plane with 45º of external rotation

For the sake of comparison, Figures S5 and S7 present the contact pressures for the different bone graft positions under a similar scale. For a more detailed evaluation of the results obtained for each bone graft position, Figures S6 and S8 present the same contact pressures considering scales adjusted for each model.

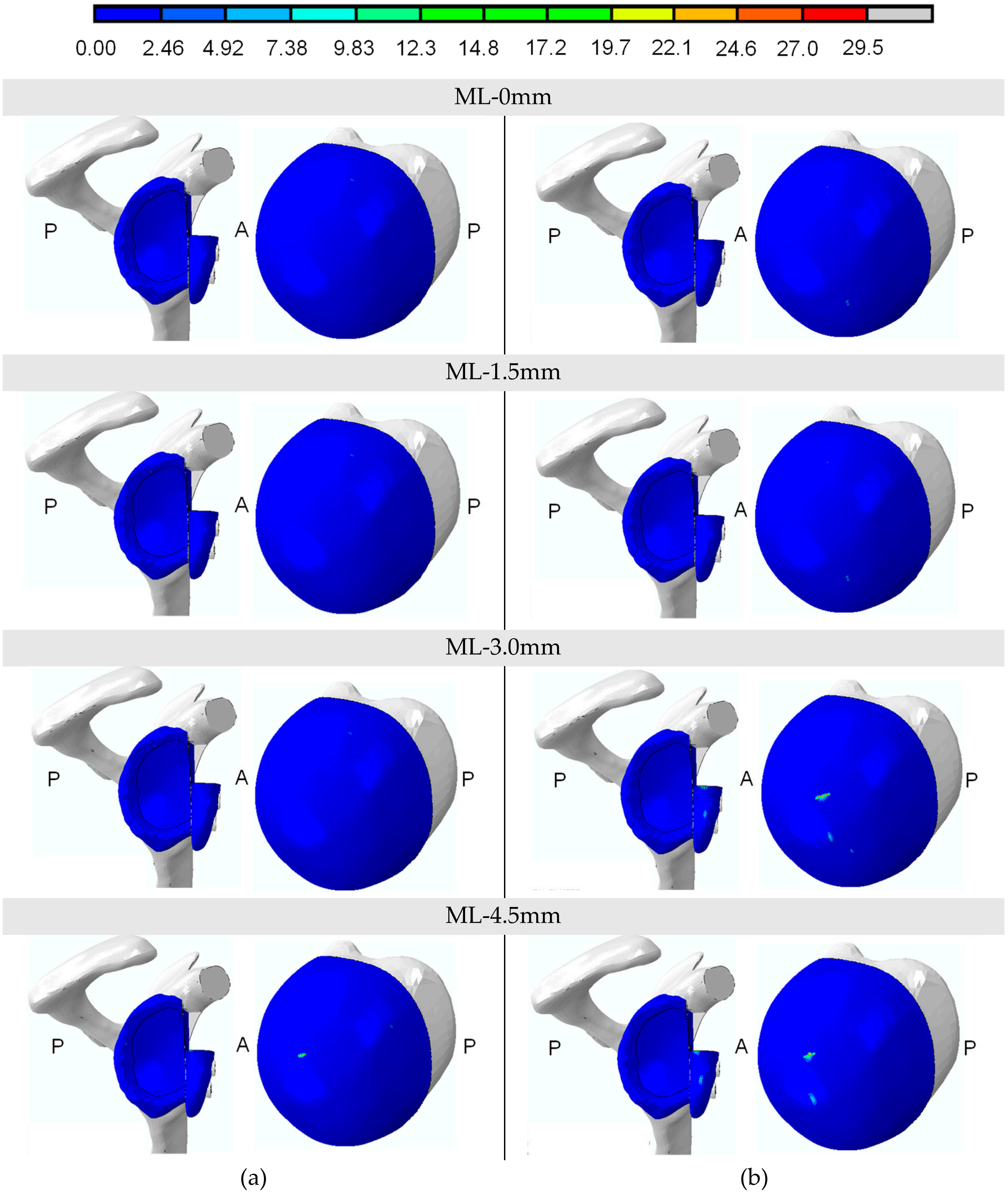

Figure S5 – Contact pressure distribution, in MPa, after the Latarjet procedure for two anterior translation points: (a) at the beginning, and (b) at the peak translation force. Results correspond to the 60º shoulder abduction in the scapular plane with 45º of external rotation under a 50 N compressive force. A similar scale is considered for all bone graft positions.

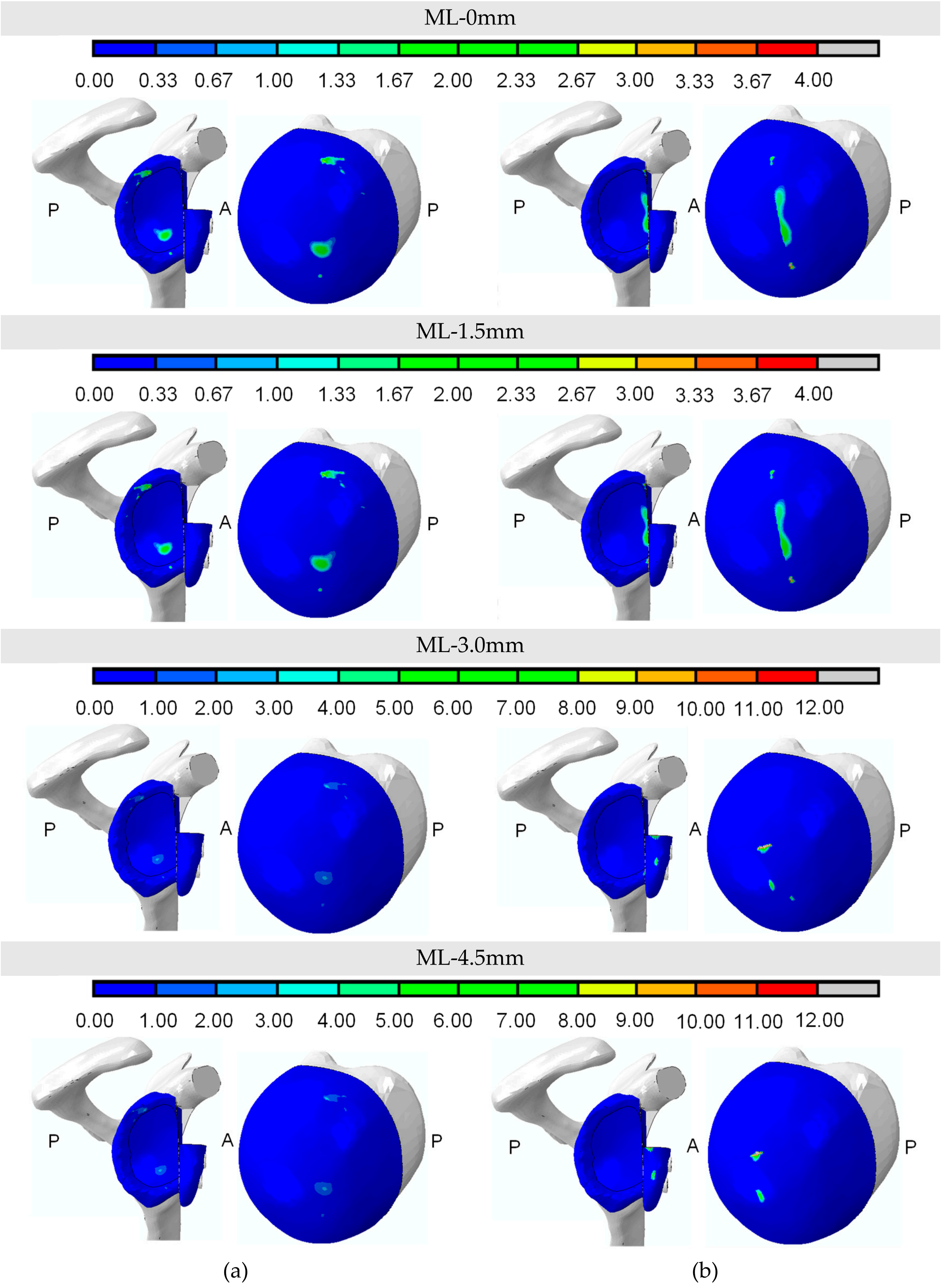

Figure S6 – Contact pressure distribution, in MPa, after the Latarjet procedure for two anterior translation points: (a) at the beginning, and (b) at the peak translation force. Results correspond to the 60º shoulder abduction in the scapular plane with 45º of external rotation under a 50 N compressive force. Each bone graft position presents its own scale.

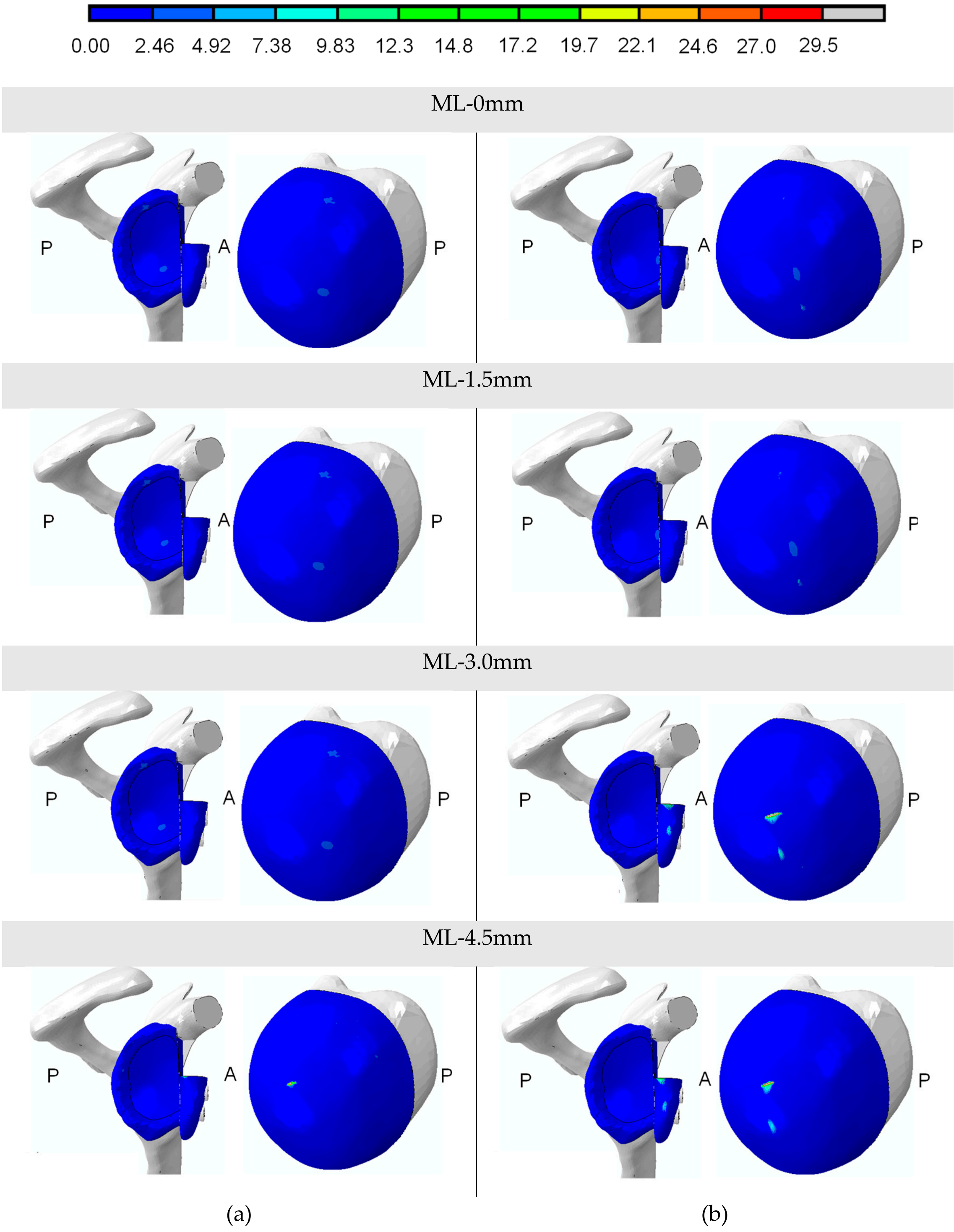

Figure S7 – Contact pressure distribution, in MPa, after the Latarjet procedure for two anterior translation points: (a) at the beginning, and (b) at the peak translation force. Results correspond to the 60º shoulder abduction in the scapular plane with 45º of external rotation under a 100 N compressive force. A similar scale is considered for all bone graft positions.

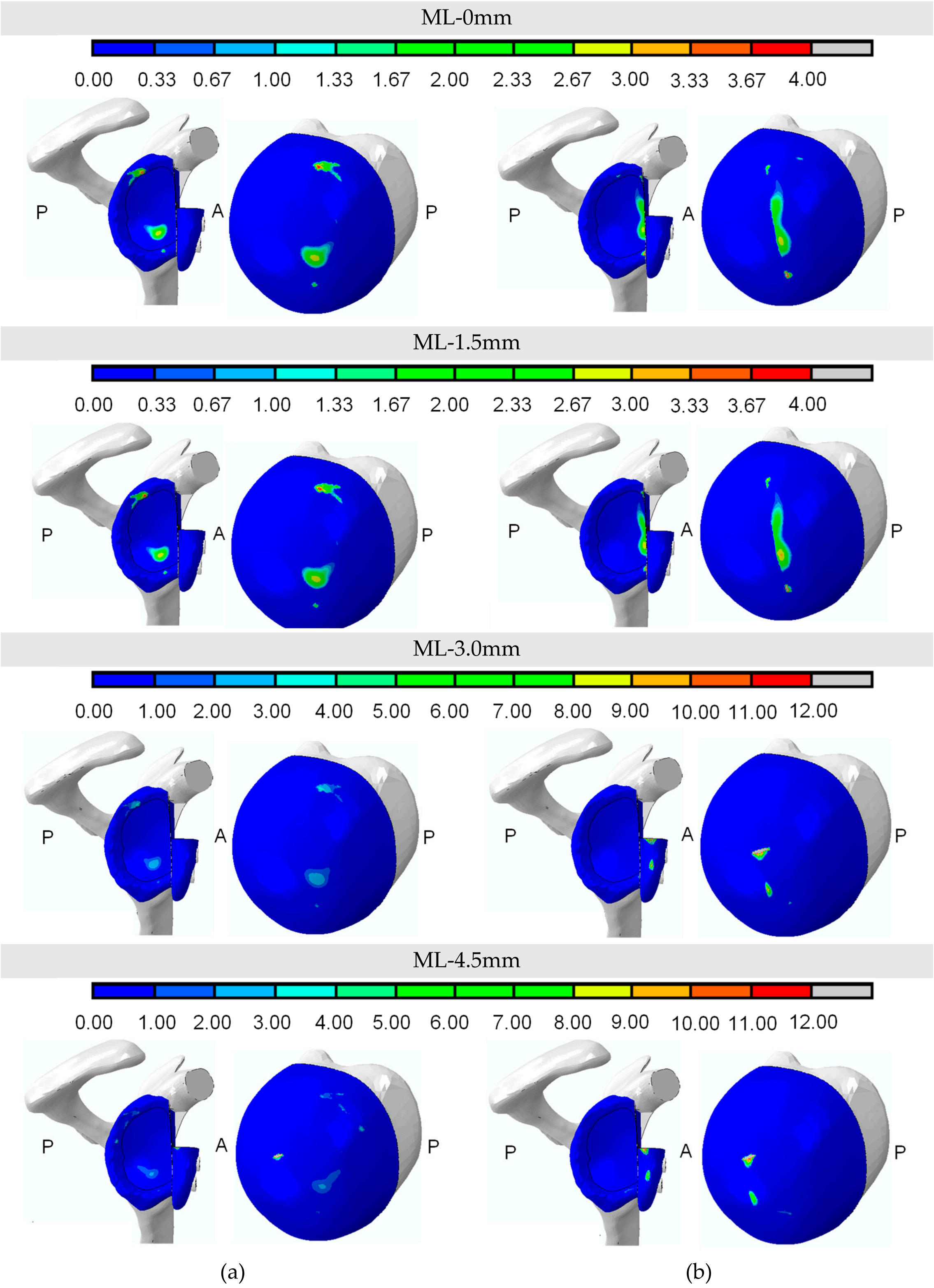

Figure S8 – Contact pressure distribution, in MPa, after the Latarjet procedure for two anterior translation points: (a) at the beginning, and (b) at the peak translation force. Results correspond to the 60º shoulder abduction in the scapular plane with 45º of external rotation under a 100 N compressive force. Each bone graft position presents its own scale.
